# Posture-induced changes in the vessels of the head and neck: evaluation using conventional supine CT and upright CT

**DOI:** 10.1038/s41598-020-73658-0

**Published:** 2020-10-06

**Authors:** Kenzo Kosugi, Yoshitake Yamada, Minoru Yamada, Yoichi Yokoyama, Hirokazu Fujiwara, Keisuke Yoshida, Kazunari Yoshida, Masahiro Toda, Masahiro Jinzaki

**Affiliations:** 1 Department of Neurosurgery, Keio University School of Medicine, 35 Shinanomachi, Shinjuku-ku, Tokyo, 160-8582 Japan; 2 Department of Radiology, Keio University School of Medicine, 35 Shinanomachi, Shinjuku-ku, Tokyo, 160-8582 Japan

**Keywords:** Physiology, Anatomy

## Abstract

Since the venous system is affected by gravity, upright computed tomography (CT) in addition to conventional supine CT has great potential for evaluating postural changes in the venous system. We evaluated the morphological differences in the head and neck vessels by performing a contrast CT study in both the supine and the sitting positions. In this study, the 20 included participants (10 men and 10 women) were healthy adults aged 30 to 55 years. The cross-sectional area of the cervical vessels, craniocervical junction veins, and intracranial vessels were obtained quantitatively. Venous sinuses and venous plexuses that were difficult to measure were evaluated qualitatively. The average change in areas from a supine to an upright posture was − 77.87 ± 15.99% (*P* < 0.0001) in the right internal jugular vein (IJV), − 69.42 ± 23.15% (*P* < 0.0001) in the left IJV, − 61.52 ± 12.81% (*P* < 0.0001) in the right external jugular vein (EJV), and − 58.91 ± 17.37% (*P* < 0.0001) in the left EJV. In contrast, the change in the anterior condylar vein (ACV) from a supine to an upright posture was approximately + 144% (*P* < 0.005) on the right side and + 110% (*P* < 0.05) on the left side. In addition, according to the qualitative analysis, the posterior venous structures including the anterior condylar confluence (ACC) of the craniocervical junction became more prominent in an upright posture. Despite these changes, the intracranial vessels showed almost no change between postures. From a supine to an upright position, the IJVs and EJVs above the heart collapsed, and venous channels including the ACCs and ACVs opened, switching the main cerebral venous drainage from the IJVs to the vertebral venous system. Upright head CT angiography can be useful for investigating physiological and pathophysiological hemodynamics of the venous system accompanying postural changes.

## Introduction

Humans spend a majority of the day in an upright position. However, diagnostic imaging devices, which are routinely used in clinical settings, are designed for a supine position instead. Therefore, the hemodynamic changes within the craniocervical area between a supine and an upright posture are not fully understood.

The human body consists of many organ systems. The development of computed tomography (CT) has enabled the systematic cross-sectional visualization of many organ systems, including the arterial system, respiratory system, biliary tract, alimentary tract, and urinary system^1–7^. However, conventional supine CT or magnetic resonance imaging (MRI) alone is inadequate for evaluating the pathophysiology of the venous system, in which the distribution of volume is affected by gravity. Therefore, in order to evaluate the effect of gravity on the venous system, it is necessary to use a diagnostic imaging technique that can image the whole body in an upright posture. Even at the time when CT was developed in 1972^8^, there was an intention to develop this modality for use in a standing position. However, this goal was not realized due to the difficulty of keeping subjects standing for the length of time it took to image the whole body. It was even more difficult with MRI because it takes longer to perform than CT. The recent improvement in CT has resulted in shorter scanning times, and the latest 320-row detector CT scanner can image the whole head within 5 s using helical mode and obtain 3-dimensional images of the whole head over time (4-dimensional imaging) using a 16-cm coverage axial scan. Recently, in collaboration with Toshiba Medical Systems (currently Canon Medical Systems), we proposed and developed an upright 320-detector row CT scanner; this scanner can obtain images of subjects in a standing or sitting position^9,10^. Using this system, we found that gravity differentially affected the volume and shape of the vena cava within the torso depending on body position^10^. However, the effect of gravity on the venous system in the head and neck is still not fully understood.

In a supine position, the internal jugular veins (IJVs) are the primary venous drain for the brain^11–13^. Several studies have recently reported that the jugular veins are collapsed and the main venous outflow from the brain occurs via the vertebral venous systems (VVS) in an upright position^13–15^. The VVS contains veins, venous plexuses, and venous sinuses that course along the entire length of the spine^13^, it is regarded as a unique, large-capacity, valveless plexiform venous network in which the flow is bidirectional^16^. The condylar veins (anterior, lateral, and posterior) and the anterior condylar confluence (ACC), which is located extracranially in front of the aperture of hypoglossal canal, represent the most important connections between the intracranial cerebral venous circulation and the VVS^17^, and have been suggested to contribute to the main outflow tract for encephalic drainage in the upright position. However, such positional changes have not been actually illustrated in the past in humans. In addition, no previous reports have revealed morphological changes in intracranial vessels including venous structures, in different postures.

The purpose of the study was to elucidate the posture-related changes in craniocervical junction venous structures, intracranial vessels and cervical vessels by obtaining contrast-enhanced CT images of healthy volunteers in both supine and sitting positions. The positional changes in vessels within the head and neck are clinically important not only for physiological cerebral venous drainage but also in pathological conditions such as ICP abnormalities, cerebrovascular disease, and tumors.

## Results

### Study population

The study participants were 10 male and 10 female volunteers. The mean (± standard deviation) age and body mass of the male volunteers were 43.6 ± 7.8 years (range, 30 to 54 years) and 62.3 ± 5.7 kg, respectively, whereas those of the females were 45.5 ± 7.2 years (range, 34 to 55 years) and 58.8 ± 11.4 kg, respectively. We confirmed that no abnormalities were noted at their most recent medical checkup, and they were not taking any oral medications.

### Cervical vessels

**Figure 1:**
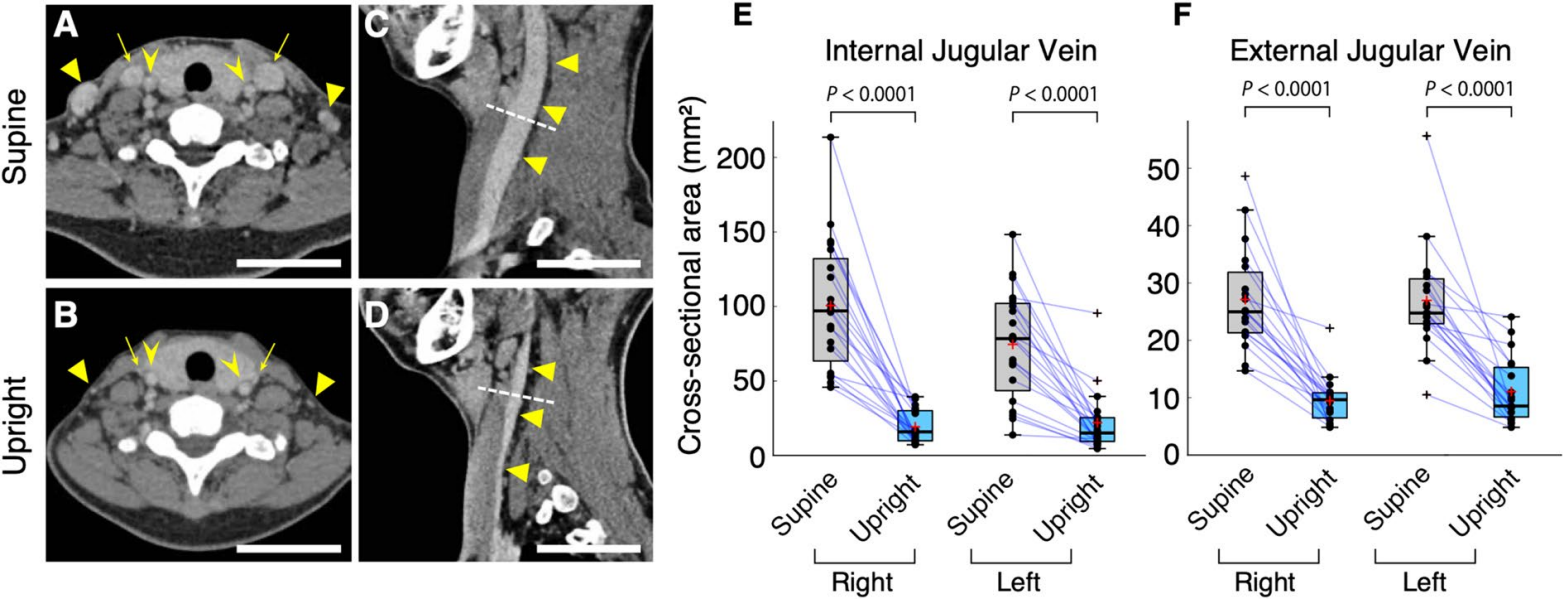
Neck veins are collapsed in an upright body position. (**A**, **B**) Axial computed tomography (CT) section from the recirculation phase. Arrows, triangles, and arrowheads show the internal jugular veins (IJVs), external jugular veins (EJVs), and internal carotid arteries, respectively. Although the internal carotid arteries hardly change in different postures, the IJVs and EJVs are significantly collapsed in an upright posture. Scale bars: 50 mm. (**C**, **D**) Sagittal CT section from the same phase. The IJVs (arrowheads) are significantly collapsed. The dashed white line indicates the level of area measurement. (**E**, **F**) The cross-sectional area of the IJVs (**E**, n = 20, two-sided paired *t*-test, right: *P* < 0.0001; left: *P* < 0.0001) and EJVs (**F**, n = 19, two-sided paired *t*-test, right: *P* < 0.0001; left: *P* < 0.0001). In the box plots, the central mark indicates the median, the red cross indicates the mean, and the bottom and top edges of the box indicate the 25th and 75th percentiles, respectively. Whiskers extend to the maximum and minimum values within 1.5 interquartile ranges below the first quartile or above the third quartile, and black crosses indicate outliers. Increases and decreases from a supine position to an upright position are colored in red and blue, respectively.

First, the postural changes in the cross-sectional area of the cervical vessels were evaluated. The internal carotid artery underwent no significant postural changes (Table S1). The IJVs and the external jugular veins (EJVs) were significantly collapsed in an upright posture (right IJVs: n = 20, average change ratio (Δ_%_) =  − 77.87 ± 15.99%, *P* < 0.0001; left IJVs: n = 20, Δ_%_ =  − 69.42 ± 23.15%, *P* < 0.0001; right EJVs: n = 19, Δ_%_ =  − 61.52 ± 12.81%, *P* < 0.0001; left EJVs: n = 19, Δ_%_ =  − 58.91 ± 17.37%, *P* < 0.0001) (Fig. 1, Table S1). Laterality was evaluated and the cross-sectional area of the IJVs was clearly superior on the right side (n = 20, *P* < 0.05) (Figure S1). The intra- and interrater reliability were substantial in all measurements (0.764–0.993) (Table S1).

### Craniocervical junction and intracranial vessels

**Figure 2:**
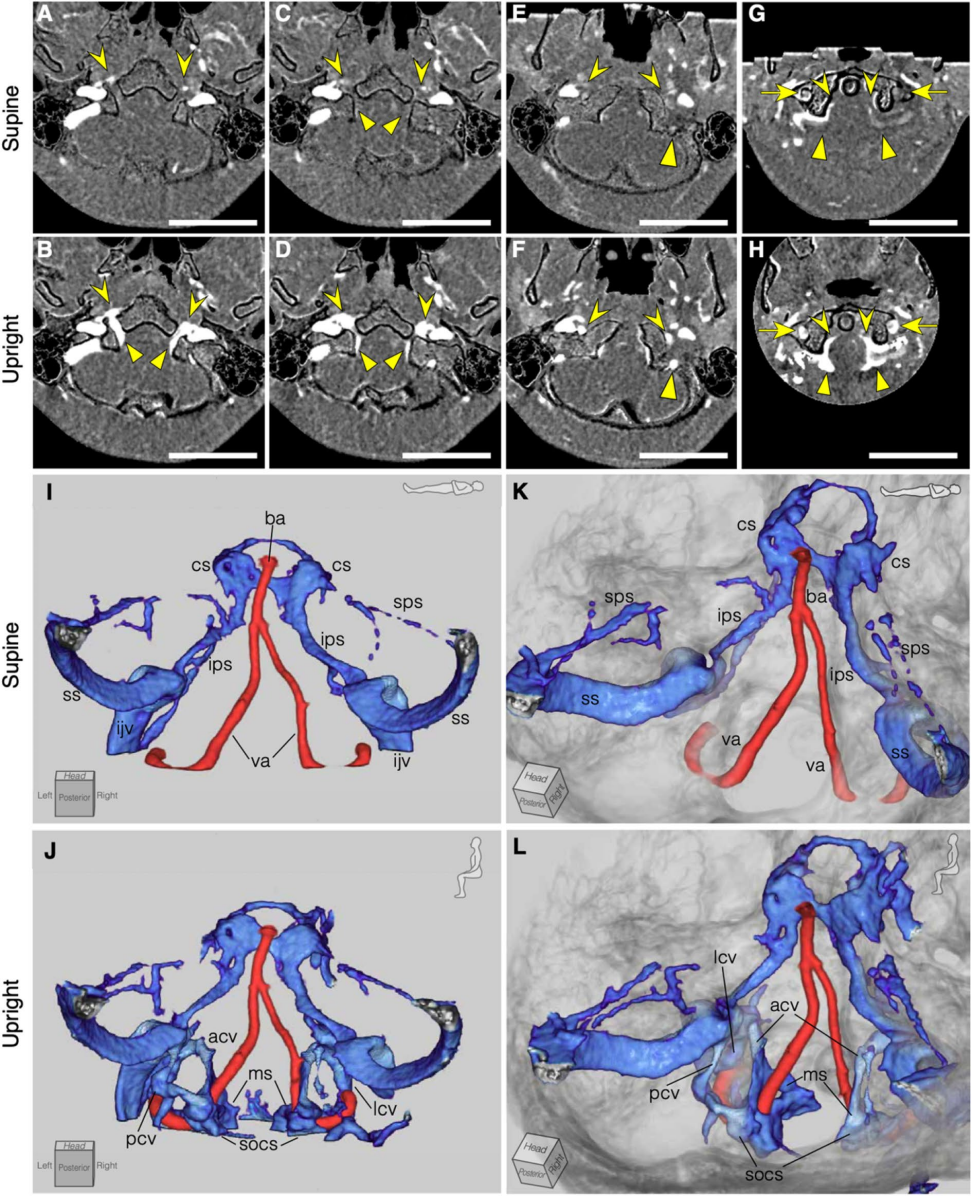
Illustrative representation of an enhanced craniocervical junction venous structure in an upright position compared with a supine position. (**A**–**H**), Axial section of bone-subtracted contrast computed tomography (CT) images. The upper column represents the supine position, and the lower column represents the upright position. Scale bars; 50 mm. (**A**, **B**) Anterior condylar confluence (ACC, arrowhead) and anterior condylar vein (triangle) are more dilated in the upright position than in the supine position. (**C**, **D**) The ACC (arrowhead) and marginal sinus (triangle) are dilated in the upright position. (**E**, **F**) The lateral condylar vein (arrow head) and posterior condylar vein (triangle) are dilated in the upright position. (**G**, **H**) The vertebral artery venous plexus (arrow), anterior internal vertebral venous plexus (arrowhead), and suboccipital cavernous sinus (triangle) become more prominent in the upright position than in the supine position. (**I**–**L**) Three-dimensional images of an illustrative case, selectively visualizing vessels in the posterior fossa and translucent cranial bone. The condylar veins, marginal sinus, suboccipital cavernous sinus become more prominent in the upright position. ba, basilar artery; va, vertebral artery; cs, cavernous sinus; sps, superior petrosal sinus; ips, inferior petrosal sinus; ss, sigmoid sinus; ijv, internal jugular vein; acv, anterior condylar vein; lcv, lateral condylar vein; pcv, posterior condylar vein; ms, marginal sinus; socv, suboccipital cavernous sinus.

**Figure 3:**
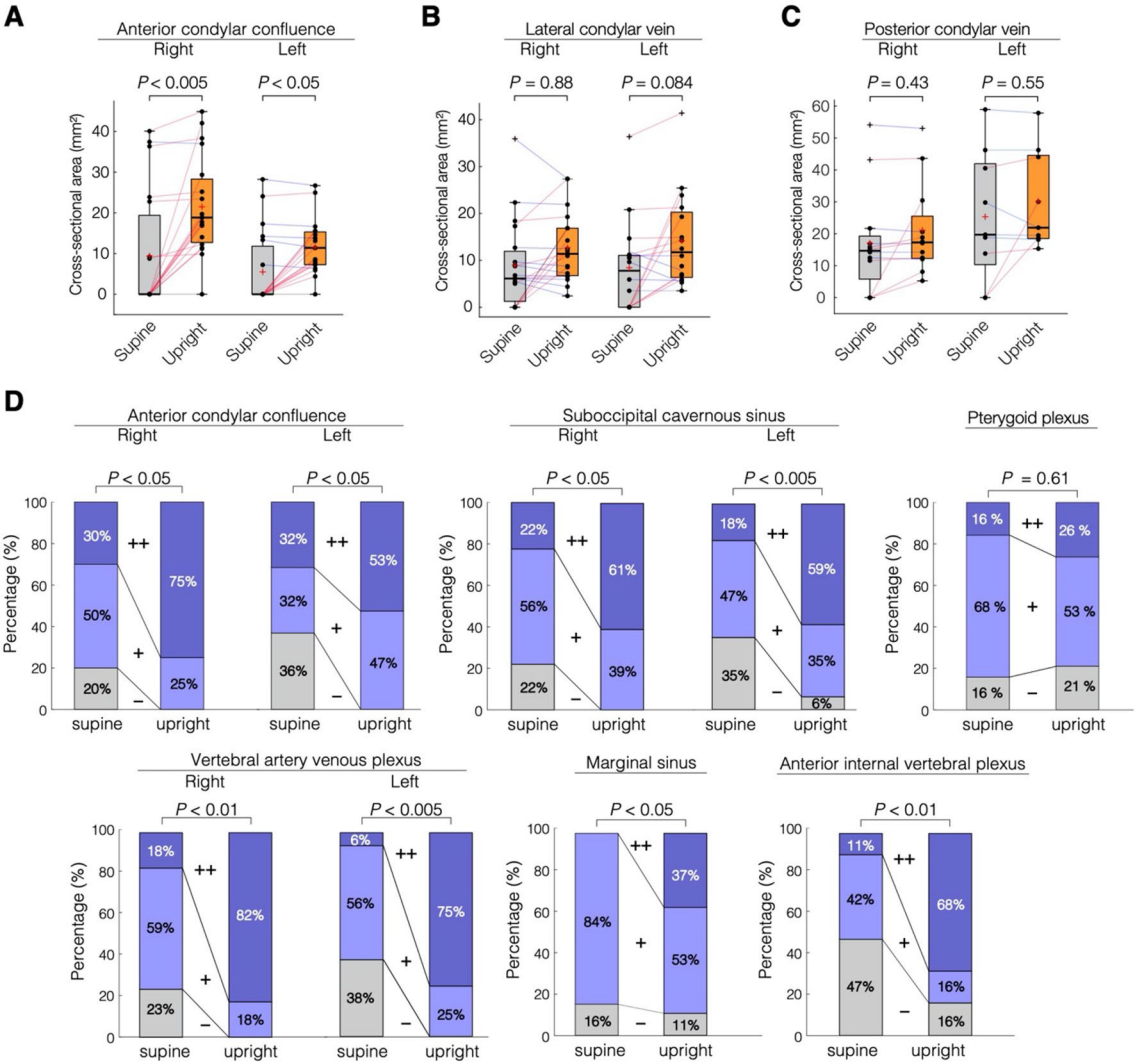
Quantitative analyses of the condylar veins and qualitative analyses of the craniocervical venous sinus and venous plexus. (**A**) The cross-sectional areas of the anterior condylar veins (**A**, n = 19, two-sided Wilcoxon signed-rank test, right, *P* = 0.00139; left, *P* = 0.0183) are significantly larger in the upright position than in supine position. The lateral and posterior condylar veins underwent no significant postural change. (**B**, Two-sided Wilcoxon signed-rank test, right: n = 19, *P* = 0.882; left: n = 16, *P* = 0.086. (**C**, Two-sided Wilcoxon signed-rank test, right: n = 12, *P* = 0.452; left: n = 9, *P* = 1.0) All *P* values were adjusted with the Bonferroni correction. In the box plots, the boxes represent the 25th-75th percentiles, the black line indicates the median, the red cross represents the mean, and the whiskers extend to the maximum and minimum values within 1.5 interquartile ranges below the first quartile or above the third quartile. Outliers are shown as black crosses. Increases and decreases from the supine position to the upright position are colored in red and blue, respectively. (**D**) McNemar–Bowker tests for correlated nominal scales were performed, and all venous structures except the pterygoid plexus showed significant changes in proportion between the supine and upright positions (right ACC: n = 20, *P* < 0.05; left ACC: n = 19, *P* < 0.05; right SOCS: n = 18, *P* < 0.05; left SOCS: n = 17, *P* < 0.005; right VAVP: n = 17, *P* < 0.01; left VAVP: n = 16, *P* < 0.005; marginal sinus: n = 19, *P* < 0.05; AIVVP: n = 19, *P* < 0.01; pterygoid plexus: n = 19, *P* = 0.61). Bonferroni-corrected post hoc McNemar tests were performed on three comparisons (−/+ , −/++, and +/++) in each vessel, and most venous structures showed significant change in the direction of distension (the direction from + to ++) from supine to upright postures (right ACC; +/++, *P* < 0.05, left ACC; −/+, *P* < 0.05, right VAVP; +/++, *P* < 0.01, left VAVP; +/++, *P* < 0.05, right SOCS; +/++, *P* < 0.05, left SOCS; +/++, *P* < 0.05, AIVVP; +/++, *P* < 0.05).

Next, the condylar veins located at the craniocervical junction and intracranial vessels were evaluated. The condylar veins are venous connections between the dural sinuses of the posterior fossa and the VVS and consists of three veins: the anterior, lateral, and posterior condylar veins. The anterior condylar vein (ACV) could be measured in 13/40 sides (33% of the sides) in the supine position and in 36/40 sides (90% of the sides) in the upright position. The lateral condylar vein (LCV) was present in 24/40 (60% of the sides) in the supine position and in 35/40 sides (88% of the sides) in the upright position. The posterior condylar vein (PCV) was present in 16/40 (40% of the sides) in the supine position and in 21/40 (53% of the sides) in the upright position. Only data in which the cross-sectional area of the target vessel could be measured in at least one supine and upright position were analyzed. Thus, 19 ACVs were analyzed on the both sides, 19 LCVs were analyzed on the right side and 16 on the left side, and 12 PCVs were analyzed on the right side and 9 on the left side. The ACVs were significantly dilated in an upright posture (right ACVs: n = 19, Δ_%_ =  + 144.86%, *P* < 0.005; left ACVs: n = 19, Δ_%_ =  + 110.69%, *P* < 0.05) (Figs. 2A,B, 3A). The LCVs and PCVs became more prominent in an upright posture, but the difference was not significant (right LCVs: n = 19, Δ_%_ =  + 42.06%, *P* = 0.882; left LCVs: n = 16, Δ_%_ =  + 68.57%, *P* = 0.084; right PCVs: n = 12, Δ_%_ =  + 17.39%, *P* = 0.426; left PCVs: n = 9, Δ_%_ =  + 30.56%, *P* = 0.546) (Fig. 3B,C). Regarding the intracranial vessels, no significant changes were found in the size of the intracranial arteries, veins, or venous sinuses (Tables 1, 2, 3). Laterality was found in several vessels; the sigmoid sinus (SS) and inferior petrosal sinus (IPS) were right-side dominant both in the supine and upright positions (SS; n = 18, *P* < 0.05, IPS; n = 14, *P* < 0.05), and the ACVs were right-side dominant only in the upright position (n = 11, *P* < 0.001) (Figure S1). The intra-observer and inter-observer reliability were substantial in all measurements (0.715–0.994) (Tables 1, 2, 3).

**Table 1:** Comparisons of areas in intracranial arteries in two positions.

Vessels	Side	Posture	N	Area (mm^2^)^a^	Change ratio (%)	95% CI of difference (mm^2^)^b^	Adjusted*P* value^c^	Intrarater reliability/interrater reliability
Internal carotid artery	Right	Supine	20	8.00 ± 1.88	+ 4.41	− 0.66 to 0.18	1.0	0.880/0.851
		Upright	20	8.25 ± 1.73				0.741/0.715
	Left	Supine	20	8.10 ± 1.85	+ 2.79	− 0.76 to 0.18	1.0	0.772/0.787
		Upright	20	8.39 ± 2.40				0.943/0.921
Middle cerebral artery	Right	Supine	20	5.00 ± 1.09	+ 5.47	− 0.51 to 0.12	1.0	0.934/0.928
		Upright	20	5.20 ± 1.02				0.848/0.818
	left	Supine	20	5.03 ± 1.20	+ 5.88	− 0.61 to 0.11	1.0	0.871/0.918
		upright	20	5.28 ± 1.34				0.722/0.739
Vertebral artery	Right	Supine	19	6.21 ± 2.49	+ 1.8	− 0.41 to 0.64	1.0	0.896/0.860
		Upright	19	6.10 ± 2.15				0.935/0.900
	Left	Supine	20	7.35 ± 2.86	− 0.53	− 0.52 to 0.65	1.0	0.980/0.978
		Upright	20	7.28 ± 2.98				0.954/0.956
Basilar artery		Supine	20	5.97 ± 1.92	+ 0.52	− 0.27 to 0.31	1.0	0.816/0.764
		Upright	20	5.95 ± 1.92				0.921/0.883

^a^The area is presented as the mean ± standard deviation.

^b^CI: confidence interval.

^c^All *P* values are adjusted for multiple comparisons.

**Table 2:** Comparisons of areas in intracranial veins in two positions.

Vessels	Side	Posture	N	Area (mm^2^)	Change ratio (%)	95% CI of difference (mm^2^)	Adjusted *P* value^c^	Intrarater reliability/interrater reliability
Cortical vein	Right	Supine	20	9.16 ± 2.67	− 4.99	0.08 to 0.80	0.133	0.876/0.825
		Upright	20	8.72 ± 2.76				0.887/0.853
	Left	Supine	20	9.27 ± 3.14	− 3.12	− 0.23 to 0.82	1.0	0.932/0.953
		Upright	20	8.97 ± 3.45				0.980/0.976
Precentral cerebellar vein		Supine	20	3.63 ± 1.05	+ 7.18	− 0.47 to 0.03	0.567	0.863/0.862
		Upright	20	3.84 ± 1.09				0.853/0.843
Superficial middle cerebral vein	Right	Supine	20	6.16 ± 1.78	+ 3.43	− 0.47 to 0.33	1.0	0.817/0.806
		Upright	20	6.22 ± 1.59				0.885/0.903
	Left	Supine	20	6.71 ± 1.98	+ 4.69	− 0.65 to 0.16	1.0	0.888/0.896
		Upright	20	6.95 ± 2.22				0.878/0.841
Internal cerebral vein	Right	Supine	20	4.64 ± 1.13	+ 6.33	− 0.45 to 0.01	0.441	0.900/0.882
		Upright	20	4.86 ± 1.01				0.874/0.882
	Left	Supine	20	4.47 ± 1.23	+ 3.91	− 0.42 to 0.27	1.0	0.902/0.948
		Upright	20	4.54 ± 1.14				0.893/0.909

^a^The area is presented as the mean ± standard deviation.

^b^CI: confidence interval.

^c^All *P* values are adjusted for multiple comparisons.

**Table 3:** Comparisons of areas in intracranial sinuses in two positions.

Vessels	Side	Posture	N	Area (mm^2^)	Change ratio (%)	95% CI of difference (mm^2^)	Adjusted *P* value^c^	Intrarater reliability/interrater reliability
Superior sagittal sinus		Supine	20	16.35 ± 5.11	+ 0.27	− 1.18 to 1.07	1.0	0.946/0.971
		Upright	20	16.41 ± 5.49				0.937/0.959
Straight sinus		Supine	20	10.74 ± 3.10	+ 3.82	− 0.85 to 0.26	1.0	0.944/0.94
		Upright	20	11.04 ± 2.93				0.957/0.986
Transverse sinus	Right	Supine	19	36.85 ± 17.45	+ 2.45	− 2.07 to 1.88	1.0	0.979/0.984
		Upright	19	36.95 ± 16.02				0.956/0.949
	Left	Supine	18	26.22 ± 10.04	+ 2.22	− 1.23 to 0.0013	0.50	0.941/0.94
		Upright	18	26.84 ± 10.31				0.979/0.962
Sigmoid sinus	Right	Supine	20	49.69 ± 16.04	+ 1.01	− 1.75 to 0.55	1.0	0.984/0.988
		Upright	20	50.29 ± 16.75				0.987/0.987
	Left	Supine	18	34.50 ± 12.88	+ 1.72	− 1.62 to 0.89	1.0	0.981/0.979
		Upright	18	34.86 ± 12.52				0.990/0.994
Superior petrosal sinus	Right	Supine	17	6.25 ± 3.21	+ 9.55	− 0.85 to 0.05	0.78	0.956/0.874
		Upright	17	6.65 ± 3.15				0.796/0.844
	Left	Supine	17	5.23 ± 1.91	+ 4.6	− 0.91 to 0.34	1.0	0.844/0.875
		Upright	17	5.51 ± 2.27				0.805/0.821
Inferior petrosal sinus	Right	Supine	15	14.08 ± 6.89	+ 3.13	− 1.19 to 0.73	1.0	0.950/0.973
		Upright	15	14.31 ± 6.93				0.922/0.982
	Left	Supine	13	11.07 ± 3.85	+ 2.7	− 1.03 to 0.52	1.0	0.896/0.904
		Upright	13	11.32 ± 4.13				0.966/0.941

^a^The area is presented as the mean ± standard deviation.

^b^CI: confidence interval.

^c^All *P* values are adjusted for multiple comparisons.

### Venous plexuses and sinuses

Finally, venous plexuses and sinuses that were difficult to measure quantitatively, were evaluated qualitatively. They were analyzed by visual reference and were classified as having poor (−), intermediate (+), or abundant (++) venous blood flow. The intracranial venous sinuses, i.e., the cavernous sinus and basilar plexus, had no apparent postural change in any of the volunteers. The pterygoid plexus located in the infratemporal fossa showed the following variety of patterns in the supine and upright posture, with no significant postural change in the proportional size by the McNemar–Bowker test (n = 19, *P* = 0.607): 10/19 (53%) had no change, 5/19 (26%) became more prominent from a supine posture to an upright posture, and 4/19 (21%) became less noticeable (Fig. 3D). The craniocervical junction venous plexuses and sinuses, including the ACCs, vertebral artery venous plexuses (VAVPs), suboccipital cavernous sinuses (SOCSs)^18^, marginal sinuses (MSs), and anterior internal vertebral venous plexuses (AIVVPs) became more prominent in an upright posture and had significant postural changes in proportional size (right ACCs: n = 20, *P* < 0.05; left ACCs: n = 19, *P* < 0.05; right VAVP: n = 17, *P* < 0.01; left VAVP: n = 16, *P* < 0.005; right SOCS: n = 18, *P* < 0.05; left SOCS: n = 17, *P* < 0.005; MS: n = 19, *P* < 0.05; AIVVP: n = 19, *P* < 0.01) (Figs. 2C–L, 3, Table S2). Bonferroni-corrected post-hoc McNemar tests were applied to three comparisons (−/+, −/++, and +/++), and all venous structures except the left ACC and MS showed significant changes in the direction of distension (the direction from + to ++) from a supine posture to an upright posture. The left ACC showed a significant change from − to +. The intra- and interrater reliability were substantial in all measurements (0.60–1.00) (Table S2).

## Discussion

In this study, we demonstrated the following three results. First, cervical veins (IJVs and EJVs) collapsed in an upright posture. Second, craniocervical junction venous structures, which flow just downstream of the outlet of the intracranial space and connect to the VVS, became more prominent in the upright position, in contrast to the more anteriorly located jugular system. Third, intracranial vessels, including arteries, veins, and venous sinuses, did not show major postural changes in contrast to venous structures in the neck and craniocervical junction.

Previous reports using devices such as echography, upright MRI, and angiography have also shown that the cervical IJVs and EJVs are collapsed in an upright posture^14,19–24^. The pressure in the IJVs is negative in this posture because they are positioned above the heart, which causes these thin-walled vessels to collapse^13^. Similar postural changes are also observed in the superior vena cava, which is also located above the heart^10^. From the perspective of cerebral venous drainage, the IJVs are the main outflow route for the cerebral blood in a supine posture. In contrast, the IJVs collapse in an upright posture and the VVS becomes the main cerebral blood outflow route in this position^13,24,25^. Although the vertebral venous plexus in the cervical portion was not investigated in this study, an upright MRI study showed that the vertebral venous plexus at the cervical level was prominent in an erect posture^23^. Therefore, the vertebral venous plexus, which courses along the entire length of the spine, plays a role in the main venous outflow or large-capacity venous reservoir in an upright posture.

**Figure 4:**
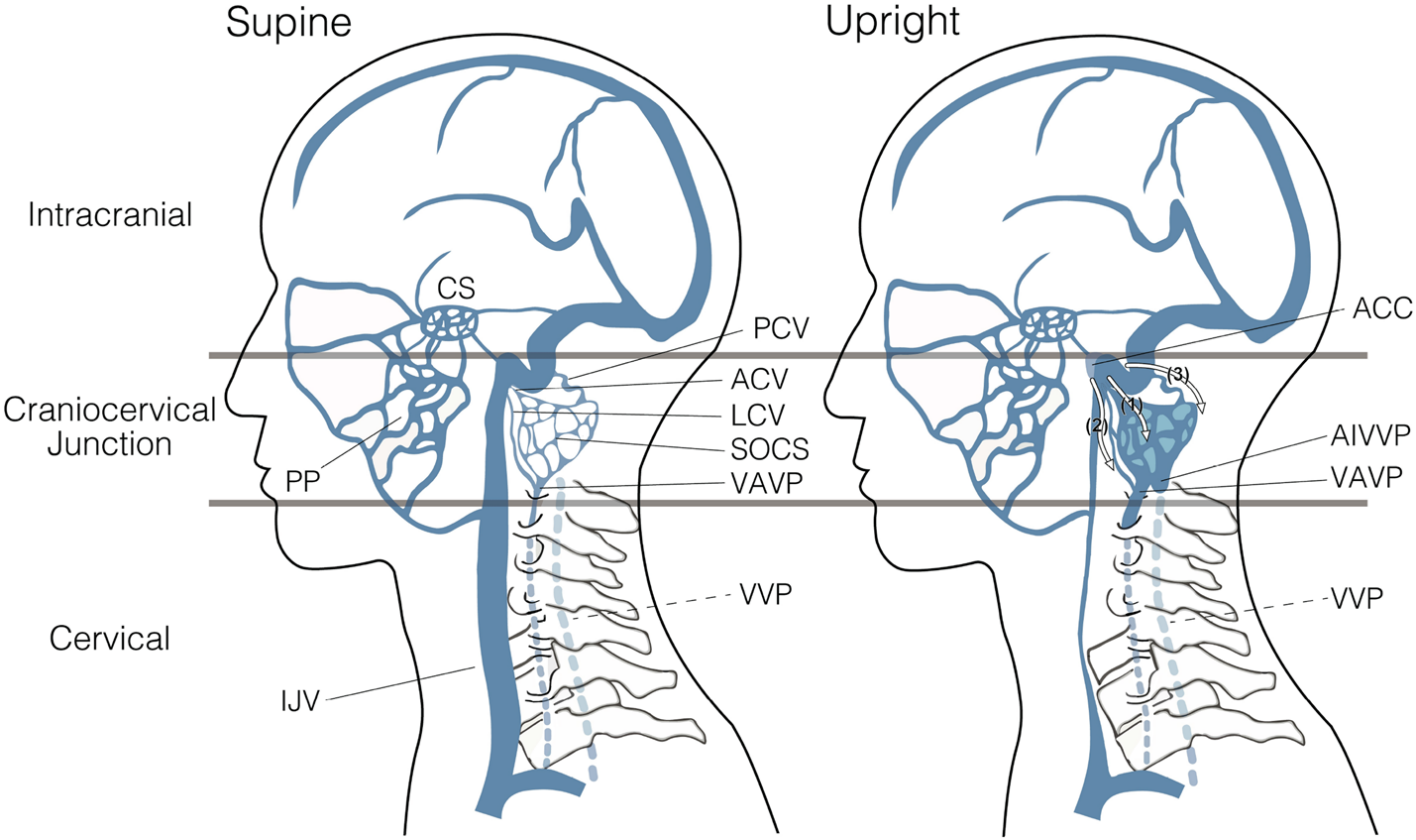
Summary of the positional changes in craniocervical venous structure between supine and upright posture. In the cervical region, the internal jugular vein (IJV) significantly collapses. In the craniocervical junction, the IJV shrinks in an upright posture; in contrast, the anterior condylar vein (ACV), anterior condylar confluence (ACC), and vertebral venous system—including the suboccipital cavernous sinus (SOCS), vertebral artery venous plexus (VAVP), and anterior internal vertebral venous plexus (AIVVP), as described in the figure—were enlarged in an upright posture. The following three venous routes become more prominent in the upright position: (1) the ACV, originating from the ACC and draining into the SOCS, VAVP, and AIVVP; (2) the LCV, originating from the ACC and draining into the SOCS, VAVP, and AIVVP; (3) the PCV, originating from the ACC or jugular bulb and draining into the SOCS, as represented by numbers in the figure. As opposed to those venous structures, the pterygoid plexus (PP), located anteriorly, does not undergo consistent changes depending on posture. In the intracranial space, the venous structure undergoes almost no change between postures. The vertebral venous plexus (VVP) at the cervical level, which could not be evaluated in this study, is shown as a dotted line.

The present study showed that the downstream ACCs, including ACVs, VAVP, SOCS, MS, and AIVVP, became more prominent in an upright posture. Although several authors have already reported that the VVS is a major venous outflow tract in an upright posture, all of these studies have been conducted in the cervical portion^13,14,17,22,23^. Therefore, this is the first study to show that the posterior venous system of the craniocervical junction was dilated in an upright position, whereas the LCVs and PCVs did not show a statistically significant change with posture. However, both upstream and downstream of the LCVs and PCVs were revealed to be more prominent in an upright posture than in a supine posture. Moreover, despite not reaching significance, their average cross-sectional areas were greater in an upright position. Accordingly, it is conceivable that the draining pathway through from the ACCs to the VVS became more enhanced in an upright posture, and the following three venous routes were considered to be more prominent in the upright position: (1) the ACVs, which originate from the ACCs, pass through the hypoglossal canal and drains into the MS, SOCS, VAVP, and AIVVP; (2) the LCVs, which originate from the ACCs and run posterolaterally to join the SOCS, VAVP, and AIVVP; and (3) the PCVs, which originate from the ACC or jugular bulb, pass through the posterior condylar canal and drain into the SOCS (Fig. 4). The ACC is located extracranially in front of the aperture of the hypoglossal canal, and its major tributaries are the condylar veins, IPS, and IJV^17^. By anastomosis with many surrounding venous structures, the ACC plays an important role as a crossroads for switching venous outflow routes between a supine position and an upright position. On the other hand, positional changes were not observed in the pterygoid plexus, which communicates from the facial vein, cavernous sinus, and inferior ophthalmic vein and drains into the EJVs in the supine position^26^. Venous blood in the pterygoid plexus perfuses primarily to the EJV in a supine position, but it may perfuse to an alternative site in an upright position because the EJV is collapsed.

Conventional MRI has a high spatial resolution, and has been reported in the past to detect differences in the cross-sectional area of intracranial vessels of approximately 1 mm^2^^27–29^. The voxel size of the 3 T MRI used in the report is 0.4 × 0.4 × 2 mm^3^ (0.32 mm^3^), and that of the CT used in this study is 0.46 × 0.46 × 0.5 mm^3^ (0.11 mm^3^); therefore, the upright CT has a higher spatial resolution than MRI. In the past, a few studies have been reported on the morphological investigation of intracranial vessels in an upright posture by using upright MRI. All of the upright MRI studies reported to date have used 0.5 or 0.6 T MRI^22–24,30^, and the spatial resolution is inadequate to measure the thin intracranial vessels. Even if CT and MRI could yield images with the same spatial resolution in the upright position, CT still has an advantage, namely that it takes less time to scan than MRI. It is more difficult to keep the body still during scanning in the upright position than in the supine position. Therefore, CT may be more suitable than MRI as shorter imaging times are better for reducing motion artifact in the upright position. On the other hand, MRI has a higher contrast resolution than CT; thus, it can generally reveal intracranial anatomy in greater detail than CT. For example, MRI would be by far the best way to observe spinal fluid dynamics^31,32^. If the body can be immobilized over a long imaging period, the development of high-resolution upright MRI will provide many new insights.

To the best of our knowledge, this is the first study to examine postural changes in intracranial vessels in humans by using upright contrast-enhanced CT. Our findings suggest that the intracranial vessels maintain homeostasis, at least morphologically, between different postures in healthy humans. In terms of homeostasis, ICP and the intracranial venous system are closely related. Postural effects on the cervical IJV are thought to be involved in controlling the postural changes in ICP^33–35^. Hydrostatic gradients in the venous system are the key factor for the postural ICP changes, and the collapsing of the IJVs in an upright posture hydrostatically isolates the dural sinus pressure from the central venous pressure, preventing ICP from falling far below zero in an upright posture^19,36,37^. In normal ICP patients and healthy volunteers, ICP was reportedly higher in a supine posture than in a sitting posture (an average difference in ICP was reportedly approximately 10 mmHg)^19,38^. The subjects in the present study were healthy volunteers and were assumed to have normal ICPs; therefore, our results suggest that intracranial venous structures remain constant in individuals with a normal ICP. On the other hand, positional changes in the intracranial venous system may be observed if ICP is abnormally altered in a positional manner. Intracranial hypertension and hypotension reportedly relate to stenosis and distension of cerebral venous sinuses, respectively. Cerebral venous stenosis has been proposed to be one of the causes of intracranial hypertension^39^. Venous hypertension in venous sinus stenosis occurs because of the resistance of venous outflow at the stenotic segment, which leads to an increase in ICP causing further venous sinus stenosis from external compression^39,40^. In contrast, in patients with intracranial hypotension, the venous sinuses are enlarged to compensate for the loss of intracranial cerebrospinal fluid volume^41^. In patients complaining of orthostatic headache and suspected cerebrospinal fluid hypovolemia, upright CT may reveal dilated intracranial venous structures compared with the supine position. Therefore, upright CT has the potential to increase the detection sensitivity for intracranial hypotension. Further study of upright CT for patients with such diseases is needed.

Asymmetrical venous drainage from the brain has been reported previously. The transverse sinus and IJV are often right-side dominant^42–45^, consistent with the results of the present study. However, there have been no reports of the ACV that show a clear laterality^46^. In the present study, no laterality was found in the ACV in the supine position, but the laterality became apparent in the upright position. This is because the venous channel was opened in the upright position; therefore, the ACV could be identified more clearly than in the supine position. It is not clear why these venous structures were often right-side dominant. The cause of IJV asymmetry has been proposed as a relationship between IJV laterality and handedness^47^, and the reason for transverse sinus asymmetry has been thought to be because the right transverse sinus originates from a prolonged superior sagittal sinus^42^. From these reports, it is easy to accept that the IPS and ACV were right-side dominant in line with these trends in other larger venous structures.

We acknowledge that there are several limitations to this study. First, the power of the present study to detect a significant postural difference in the PCVs is relatively small due to its small analyzable sample size. Second, cervical vessels, especially the vertebral venous plexus, need to be studied further in an upright position. In the present study, it was difficult to identify the internal and external vertebral venous plexus adjacent to the vertebral bones because we performed a helical single-shot scan in the recirculation phase. Third, blood velocity or blood flow in an upright position, which is essential for understanding cerebral hemodynamics, could not be evaluated in this study. Although we could not translate our results of morphological changes directly into blood flow or blood volume, these results are assumed to largely reflect hemodynamics in the head and neck.

Venous structure in the head and neck showed different postural changes: cervical veins collapsed, craniocervical junction venous structures connecting with the VVS became more prominent, and intracranial vessels hardly changed in an upright posture compared with a supine posture. From a supine to an upright position, the IJVs and EJVs above the heart collapsed, and venous channels including the ACCs and ACVs opened, switching the main cerebral venous drainage from the IJVs to the VVS. Upright head CT angiography can be useful to investigate hemodynamic changes of the venous system accompanying postural change to understand not only normal physiology but also pathophysiology.

## Materials and methods

### Statement

The present study was approved by the Keio University School of Medicine Ethics Committee, and written informed consent was obtained from the participants [UMIN Clinical Trials Registry (UMIN-CTR): UMIN000032999] (date of registration 14/06/2018). All methods were carried out in accordance with relevant guidelines and regulations.

### Study population

Three men and 3 women in the fourth decade, the same numbers in the fifth decade, and 4 men and 4 women in the sixth decade of life were enrolled in the present study. All participants understood the purpose of the study and cleared the following exclusion criteria: (1) confirmed or suspected pregnancy at the time of the study; (2) asthma or a history of asthma, (3) previous allergic reaction to the contrast materials, (4) abnormal renal function (estimated glomerular filtration rate < 60 ml/min 1.73 m^2^), (5) history of any clinical disorder, and (6) inability to maintain a stable sitting position. CT scans were performed from August 25, 2018, to February 15, 2019.

### Protocol for CT scans

All participants underwent both supine and sitting CT on the same day with an interval of one hour between scans. The orders of scans, i.e., beginning with supine CT or sitting CT, was prospectively randomized in a 1:1 ratio. Upright CT (prototype TSX-401R, Canon Medical Systems, Japan) was developed based on 320-detector row CT (Aquilion ONE, Canon Medical Systems, Japan). Upright CT also has 320-detector rows, and its performance is comparable to that of conventional supine CT as described previously^9,10^. Thus, both conventional CT and upright CT machines enable continuous or intermittent (repeated) axial scans covering 16 cm per rotation at the same position using 320 detector rows (4D-scan) and helical scans using 80 detector rows.

First, an intravenous line was inserted in the right median cubital vein. Next, a test bolus scan was performed at the level of the carotid bulb to determine the optimal timing of dynamic scans using a 10-ml intravenous injection of nonionic contrast material (OYPALOMIN, iopamidol, Fuji Pharma Co., Ltd., Tokyo, Japan) at a rate of 5 ml/s, followed by a 20-ml injection of saline. Four-dimensional CT angiography (4D-CTA) of the whole head covering a 16-cm length using 320-detector row mode was obtained after a 50-ml bolus injection of contrast material at a rate of 5 ml/sec, followed by a 20-ml injection of saline. 4D-CTA scans consisted of an unenhanced volume scan (scan time: 1 s) 4 s prior to the arrival of contrast material to generate a mask data set for subsequent bone subtraction, followed by intermittent volume scans timed at 0, 2, 4, 6, 8, 10, 12, 14, 16, 18, 20, 23, 26, 29, 32, and 60 s from the arrival of the contrast material, as determined from the test bolus scan. Finally, helical scanning from head to neck in 80-detector mode was performed at a 90-s delay (delayed helical scan). All scans were obtained with a slice thickness of 0.5 mm and a field of view of 24 cm. The voxel size was 0.46 × 0.46 × 0.5 mm^3^ (0.11 mm^3^). The other scan parameters were as follows: tube voltage of 80 kV, tube current of 350 mA for mask scans, 150 mA for intermittent scans, and rotation speed of 1.0 s for 4D-CTA scans; tube voltage of 100 kV, image SD 12 for a 5-mm slice thickness (maximum 300 mA, minimum 50 mA), and rotation speed of 0.5 s for delayed helical scans. All participants were instructed to breathe naturally, avoid holding their breath, and close their eyes during each scan.

### Data analysis

All CT images were analyzed using the 3-dimensional image analysis system volume analyzer SYNAPSE VINCENT (Fujifilm Medical Co., Ltd., Tokyo, Japan). All measurements were performed in a blinded and randomized manner.

On measurements of cervical vessels, the cross-section of the target vessel was obtained and the area was measured by tracing the edges of the vessels freehand. The cross-sectional areas of the internal carotid artery, IJV, and EJV were measured. The area of the internal carotid artery was measured at the point above the bifurcation of the common carotid artery, and those of the IJV and EJV were obtained at the level of the midcervical portion (Figure S2).

When measuring the intracranial and craniocervical junction vessels, we began by reviewing all multiphase contrast-enhanced images and selecting the appropriate images from each posture in which the CT value in the target vessel was highest. To ensure that exactly the same point of the same vessel was measured in different positions, selected CT images in each posture were almost entirely merged by using the similarity measures for image registration^48^, and a cross-section of the target vessel was obtained. The maximum CT values in the target vessels were significantly different between the supine and upright postures; therefore, we adopted the following semiautomatic measurement methods instead of the freehand measurement. The surrounding tissue/lumen border and the lumen/cranial bone border were defined as described previously^49,50^. The area was measured by using the acquired boundary CT values (Figure S3). Cross-sectional areas were measured in the following vessels: intracranial arteries, including the internal carotid artery, middle cerebral artery, vertebral artery, and basilar artery; veins, including cortical vein, precentral cerebellar vein, superficial middle cerebral vein, and internal cerebral vein; venous sinuses, including the superior sagittal sinus, straight sinus, transverse sinus, sigmoid sinus, superior petrosal sinus, and IPS; and craniocervical junction veins, including the ACV, LCV, and PCV (Figure S2). When a target vessel was delineated in either a supine or upright position only, the vessel was considered to be anatomically present; therefore, the measurement when it was not delineated was set to zero. On the other hand, if the target vessel was not delineated in either posture, such cases were not included in the results because it was unclear whether the target vessel was actually present. Venous structures located near the cranial base were not always anatomically present and were often not actually delineated by contrast-enhanced CT, resulting in a smaller sample size, especially for the PCV. The cross-sectional areas of vessels for which quantitative analysis was available were compared between the right and left sides both in the supine and upright positions, and the presence of laterality was analyzed.

Venous plexuses and sinuses, which were difficult to evaluate quantitatively, were analyzed by visual reference and were classified as having poor (−), intermediate (+), or abundant (++) venous blood flow. The cavernous sinus, basilar plexus, pterygoid plexus, ACC, MS, SOCS^18^, VAVP, and AIVVP were evaluated in this manner (Figure S4).

Except for condylar veins, the cross-sectional area in both the supine and upright positions could be measured, and the average change ratio was calculated according to the following formula:

$$ {\text{the}}\;{\text{average}}\;{\text{change}}\;{\text{ratio}}\,\left( \% \right) = \frac{1}{n}\mathop \sum \limits_{i = 1}^{n} \left( {\frac{{{\text{Area}}_{i\_upright} - {\text{Area}}_{i\_supine} }}{{{\text{Area}}_{i\_supine} }} \times 100} \right) $$

On the other hand, in many subjects, the condylar veins were poorly detected and could not be measured in the supine position in any phases; therefore, the measurements were set to zero. In those cases, the average change ratio could not be calculated according to the above formula. Thus, the average change ratio of areas of the condylar veins were calculated instead according to the following formula:

$$ {\text{the}}\,{\text{average}}\,{\text{change}}\,{\text{ratio}}\,\left( \% \right) = \frac{{{\text{average}}\,{\text{area}}_{upright} - {\text{average}}\,{\text{area}}_{supine} }}{{{\text{average}}\,{\text{area}}_{supine} }} \times 100. $$

### Statistics

For all 20 participants, the first analysis was performed by a general neurosurgeon with 6 years of experience. The same reader conducted a second analysis of 10 participants one month after the initial analysis to assess intrarater reliability, and vessel analysis of 10 volunteers was performed by a different general neurosurgeon with 5 years of experience to assess interrater reliability. The intra- and interrater reliability for categorical data were determined using Cohen’s κ. For continuous data, the intraclass correlation coefficient was calculated with the 2-way mixed-effects model estimating absolute agreement.

Statistical analyses were all conducted with SPSS version 25 (IBM Corp., Armonk, NY, USA). A two-sample comparison was performed using a paired *t*-test, and if the data were not normally distributed, a Wilcoxon signed-rank test was performed. Comparisons of paired categorical data, which were obtained from qualitative analyses of intracranial and craniocervical venous plexuses and sinuses, were performed by McNemar–Bowker tests followed by post hoc McNemar tests. A two-tailed *P* value of < 0.05 was considered significant. Bonferroni corrections were applied for multiple comparisons in each category of vessels.

## Supplementary information

Supplementary Information.

## Data Availability

The datasets generated during and/or analyzed during the current study are not publicly available due to the limitation that the data can be viewed only in the laboratory in our institution but are available from the corresponding author on reasonable request with the limitation of browsing in our facility.

## Abbreviations

ACC: Anterior condylar confluence
ACV: Anterior condylar vein
AIVVP: Anterior internal vertebral venous plexus
CT: Computed tomography
EJV: External jugular vein
ICP: Intracranial pressure
IPS: Inferior petrosal sinus
IJV: Internal jugular vein
LCV: Lateral condylar vein
MRI: Magnetic resonance imaging
MS: Marginal sinus
PCV: Posterior condylar vein
SOCS: Suboccipital cavernous sinus
SS: Sigmoid sinus
VAVP: Vertebral artery venous plexus
VVS: Vertebral venous system
