# Supplementary material for: Posture-induced changes in the vessels of the head and neck: evaluation using conventional supine CT and upright CT
Source: Sci Rep. 2020 Oct 6;10:16623. doi: 10.1038/s41598-020-73658-0 (PMC7538893; doi:10.1038/s41598-020-73658-0)
Supplement: Supplementary file 1 — Supplementary Information. [file 41598_2020_73658_MOESM1_ESM.docx]

**Supplementary Materials**

**Title:** Posture-induced changes in the vessels of the head and neck – Evaluation using conventional supine CT and upright CT

**Authors:**

Kenzo Kosugi, MD, PhD, Yoshitake Yamada, MD, PhD, Minoru Yamada, PhD, Yoichi Yokoyama, MD, Hirokazu Fujiwara, MD, PhD, Keisuke Yoshida, MD, Kazunari Yoshida, MD, PhD, Masahiro Toda, MD, PhD and Masahiro Jinzaki, MD, PhD

**Contents:**

Supplementary Material 1, Figure

Figure S1. Comparison of the cross-sectional area of each vessel on the right and left.

Supplementary Material 2, Figure

Figure S2. The measurement point of each vessel.

Supplementary Material 3, Figure

Figure S3. Schema of the method for measuring the areas of intracranial vessels.

Supplementary Material 4, Figure

Figure S4. Examples of qualitative analyses of venous sinuses and venous plexuses.

Supplementary Material 5, Table

Table S1. Postural differences in the cross-sectional areas of cervical vessels and craniocervical junction veins.

Supplementary Material 6, Table

Table S2. Qualitative analyses of venous sinuses and venous plexuses.

**Supplementary Material 1, Figure**

**Figure S1. Comparison of the cross-sectional area of each vessel on the right and left.**


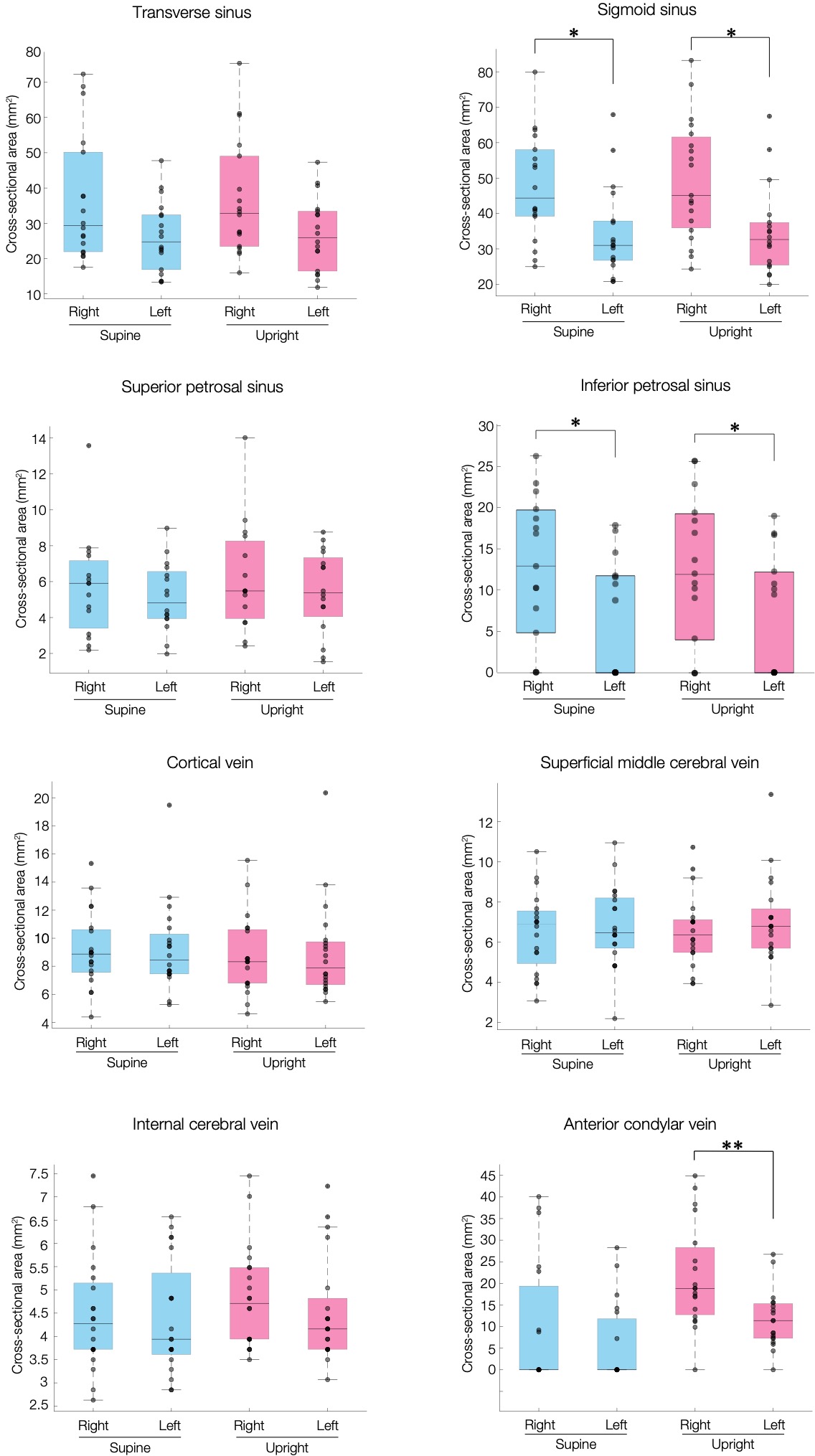


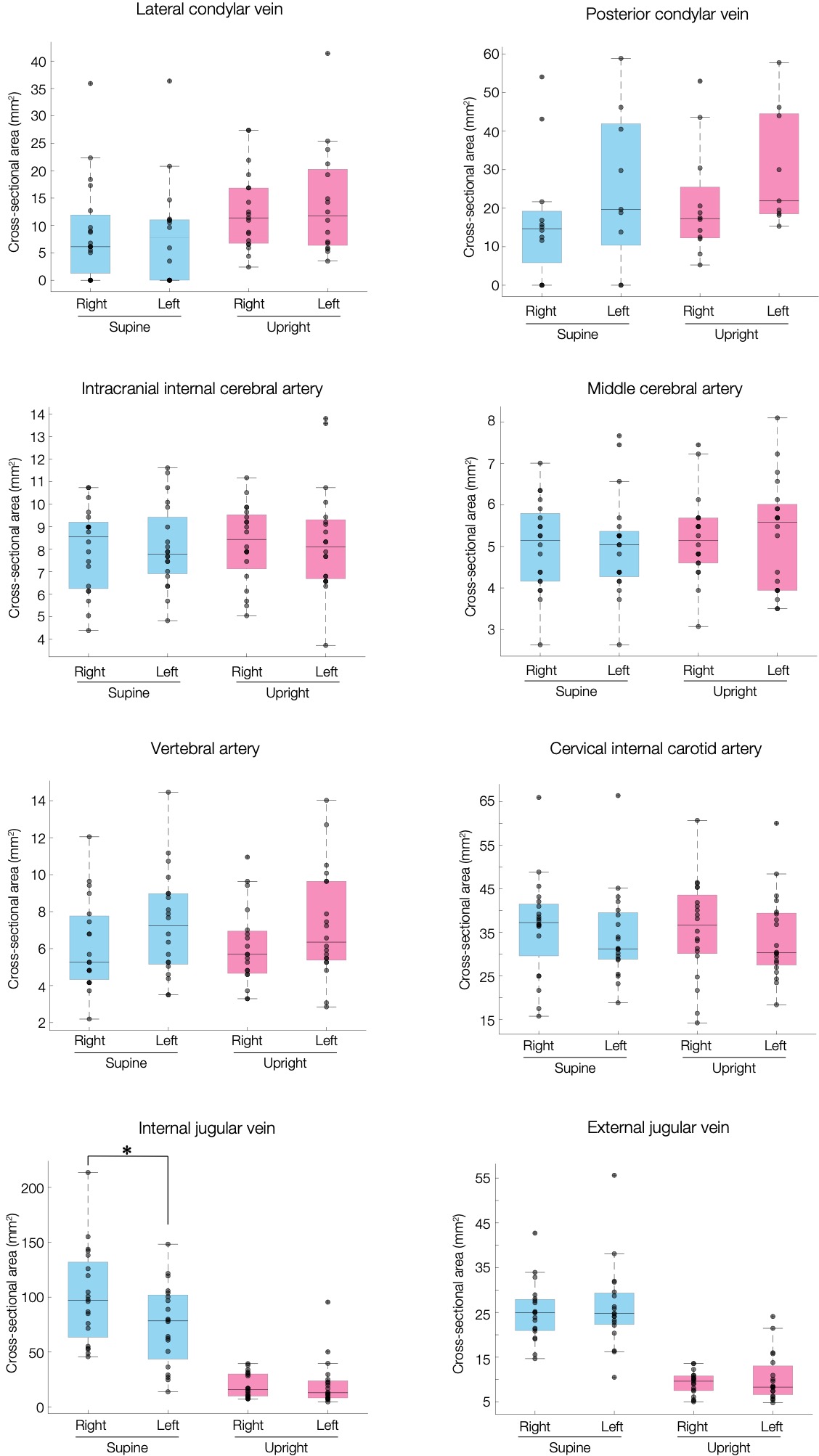


**Supplementary Material 1, Figure S1. Comparison of the cross-sectional area of each vessel on the right and left.**

The sigmoid sinus and inferior petrosal sinus are significantly larger on the right side than on the left side in both supine and upright positions. The cross-sectional area of the internal jugular vein is significantly larger on the right side than on the left only in the supine position. The anterior condylar vein is significantly larger on the right side than on the left side only in the upright position. * *P* < 0.05, ** *P* < 0.001**Supplementary Material 2, Figure**

**Figure S2. The measurement point of each vessel.**

**
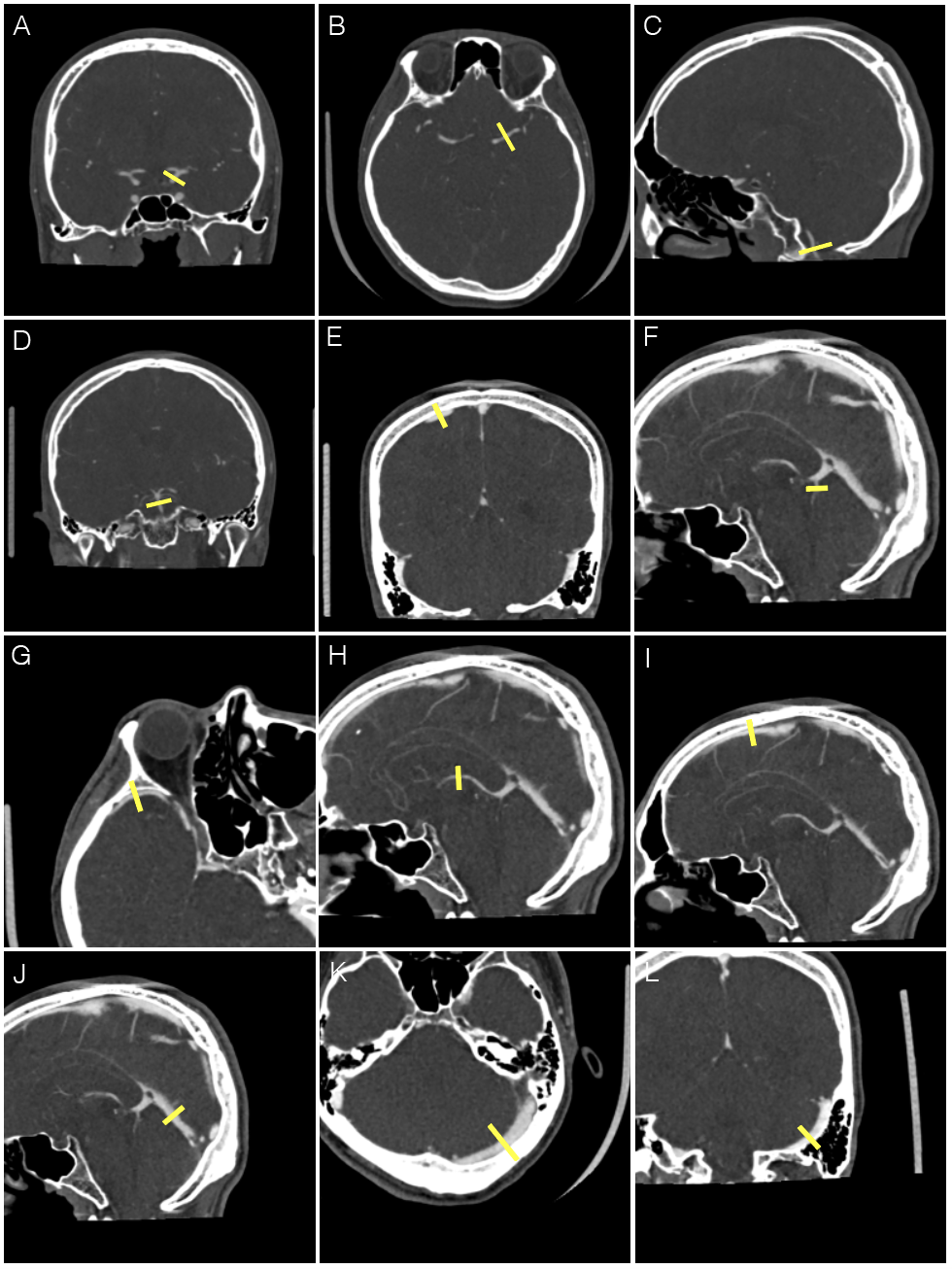
**

**
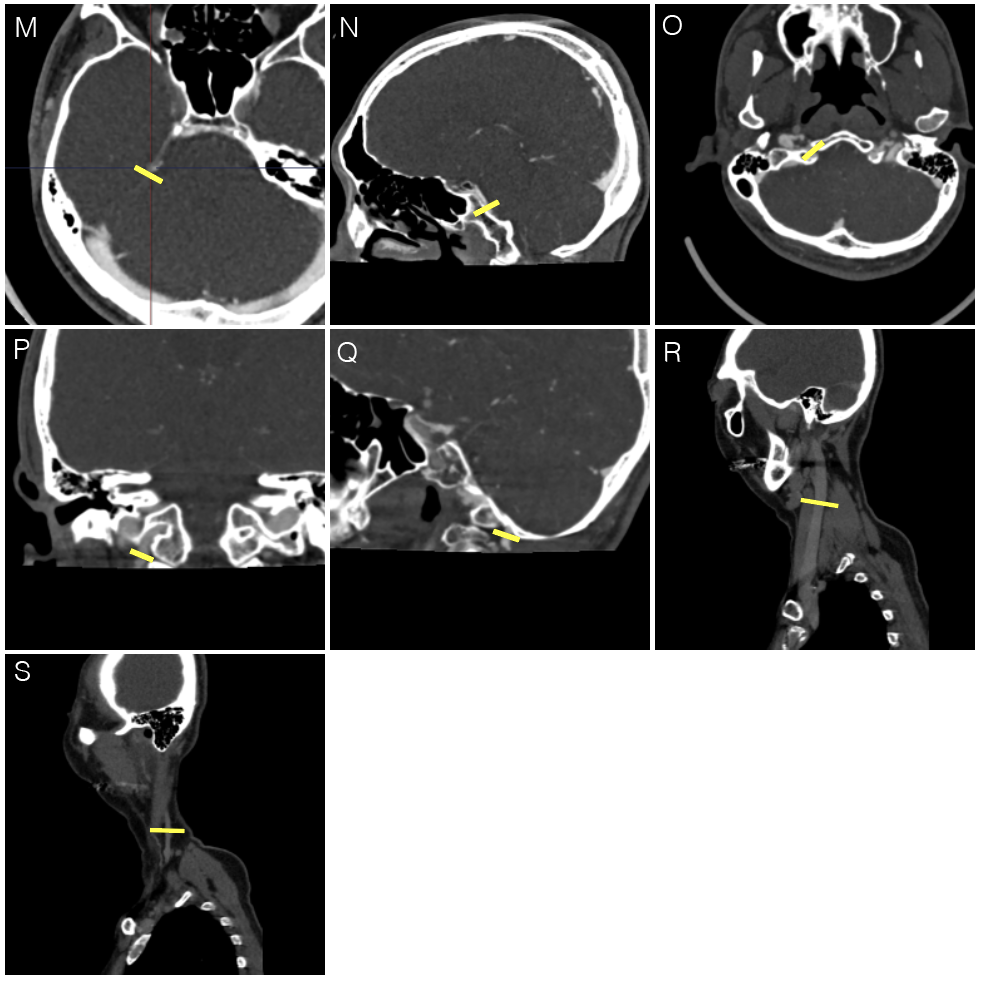
**

**Supplementary Material 2, Figure S2. The measurement point of each vessel.** **A**, Internal carotid artery, just below the bifurcation. **B**, Middle cerebral artery in the middle portion of M1. **C**, Vertebral artery, just above the foramen magnum. **D**, Basilar artery just below the point at which the superior cerebellar artery bifurcates. **E**, Cortical vein in the parietal region where it is large enough to be measured. **F**, Precentral cerebellar vein below the point where it drains into the Galenic vein. **G**, Superficial middle cerebellar vein at the point adjacent to the sphenoid ridge. **H**, Internal cerebral vein at the point where it runs horizontally. **I**, Superior sagittal sinus at the point just under the bregma. **J**, Straight sinus, middle portion. **K**, Transverse sinus, middle portion. **L**, Sigmoid sinus, middle portion. **M**, Superior petrosal sinus, middle portion. **N**, Inferior petrosal sinus at the height of the mid-clivus. **O**, Anterior condylar vein at the middle portion of the hypoglossal canal. **P**, Lateral condylar vein just under the anterior condylar confluence. **Q**, Posterior condylar vein just below its point of exit from the posterior condylar canal. **R**, **S**, Internal and external jugular veins, midcervical portion. Note that each comparison between different postures was evaluated with almost completely merged images.

**Supplementary Material 3, Figure**

**Figure S3. Schema of the method for measuring the areas of intracranial vessels.**


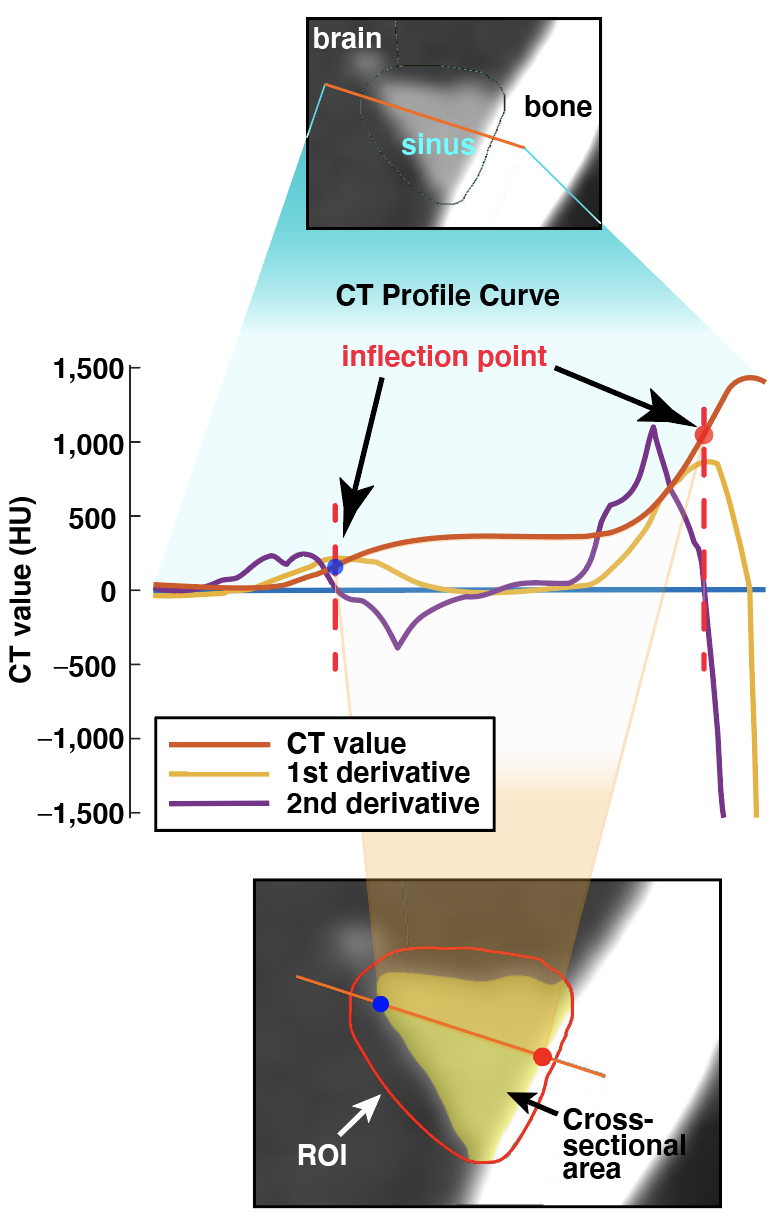
**Supplementary Material 3,** **Figure S3. Schema of the method for measuring the areas of intracranial vessels.**

A cross-section of the target vessel was obtained. The CT value profile was measured along a line through the surrounding tissue, the center of the target vessels, and any cranial bone adjacent to the vessels. The orange line indicates the CT value profile and its function. The first and second derivatives of the CT value function profiles were calculated (the yellow line shows the 1st derivative, and the purple line shows the 2nd derivative); the points (inflection points) where the first derivative reached a positive peak and the second derivative was 0 were defined as the borders between surrounding structures and the lumen border. Then, the region of interest was set around the target vessels, and the area was obtained semiautomatically from the acquired boundary CT values. The yellow area indicates the cross-section area.

**Supplementary Material 4, Figure**

**Figure S4. Examples of qualitative analyses of venous sinuses and venous plexuses.**


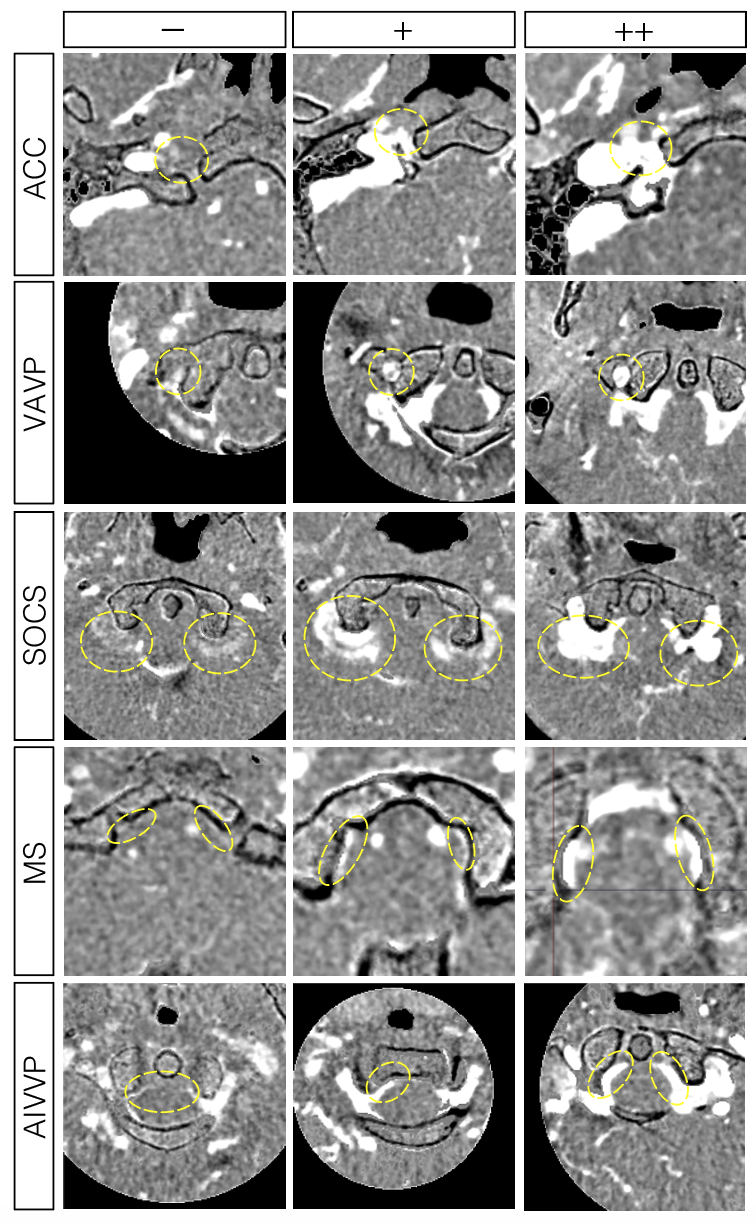


**Supplementary Material 4, Figure S4.** **Examples of qualitative analyses of venous sinuses and venous plexuses.** (−) indicates poor, (+) indicates intermediate, and (++) indicates abundant venous flow. Each venous structure is highlighted by a yellow dashed-line circle. The anterior condylar confluence (ACC) was anterolateral to the hypoglossal canal and anteromedial to the jugular bulb. The vertebral artery venous plexus (VAVP) surrounded the vertical portion of the vertebral artery in the foramen of the transverse process of the vertebra. The suboccipital cavernous sinus (SOCS) surrounded the horizontal portion of the vertebral artery. The marginal sinus (MS) arose superiorly from the anterior condylar vein and drained into the SOCS or the anterior internal vertebral venous plexus (AIVVP). The AIVVP was in the anterior epidural space of the spine.

**Supplementary Material 5, Table**

**Table S1. Postural differences in the cross-sectional areas of cervical vessels and craniocervical junction veins.**

| **Vessels** | **Side** | **Posture** | **N** | **Area (mm^2^) ^a^** | **Change ratio (%)** | **95% CI of difference (mm^2^) ^b^** | **Adjusted**  **P value ^c^** | **Intrarater reliability/Interrater reliability** |
| --- | --- | --- | --- | --- | --- | --- | --- | --- |
| Cervical internal carotid artery | right | supine | 20 | 36.28±11.45 | −1.22 | −1.31–2.43 | 1.0 | 0.993/0.985 |
|  |  | upright | 20 | 35.72±11.19 |  |  |  | 0.958/0.979 |
|  | left | supine | 20 | 34.17±10.27 | −0.89 | −0.99–1.82 | 1.0 | 0.992/0.989 |
|  |  | upright | 20 | 33.75±9.79 |  |  |  | 0.985/0.963 |
| Internal jugular vein | right | supine | 20 | 100.64±43.48 | −77.87 | 61.23–101.59 | <0.0001 | 0.955/0.938 |
|  |  | upright | 20 | 19.23±11.13 |  |  |  | 0.945/0.875 |
|  | left | supine | 20 | 74.53±36.91 | −69.42 | 37.00–69.84 | <0.0001 | 0.985/0.984 |
|  |  | upright | 20 | 21.11±21.11 |  |  |  | 0.955/0.908 |
| External jugular vein | right | supine | 19 | 25.03±6.68 | −61.52 | 12.73–18.88 | <0.0001 | 0.899/0.925 |
|  |  | upright | 19 | 9.22±2.60 |  |  |  | 0.810/0.764 |
|  | left | supine | 19 | 26.17±9.47 | −58.91 | 11.24–20.18 | <0.0001 | 0.893/0.881 |
|  |  | upright | 19 | 10.45±5.42 |  |  |  | 0.961/0.861 |

**(Continued)**

| **Vessels** | **Side** | **Posture** | **N** | **Area (mm2) ^a^** | **Change ratio (%)** | **95% CI of difference (mm^2^) ^b,d^** | **Adjusted**  **P value ^c^** | **Intrarater reliability / Interrater reliability** |
| --- | --- | --- | --- | --- | --- | --- | --- | --- |
| Anterior condylar vein | right | supine | 19 | 8.61±13.33 | +144.86 | - | < 0.05 | 0.998/0.999 |
|  |  | upright | 19 | 21.08±11.3 |  |  |  | 0.990/0.981 |
|  | left | supine | 19 | 5.50±9.24 | +110.69 | - | < 0.05 | 0.971/0.977 |
|  |  | upright | 19 | 11.58±6.65 |  |  |  | 0.947/0.954 |
| Lateral condylar vein | right | supine | 19 | 8.93±9.24 | +42.06 | - | 0.88 | 0.917/0.908 |
|  |  | upright | 19 | 12.69±7.30 |  |  |  | 0.929/0.928 |
|  | left | supine | 16 | 8.40±9.85 | +68.57 | - | 0.084 | 0.989/0.993 |
|  |  | upright | 16 | 14.17±10.07 |  |  |  | 0.962/0.958 |
| Posterior condylar vein | right | supine | 13 | 18.70±25.81 | +17.39 | - | 0.43 | 1.000/0.999 |
|  |  | upright | 13 | 21.95±25.29 |  |  |  | 0.999/0.999 |
|  | left | supine | 11 | 15.83±16.58 | +30.56 | - | 0.55 | 1.000/1.000 |
|  |  | upright | 11 | 20.67±14.55 |  |  |  | 1.000/0.998 |

^a^ The area is presented as the mean ± standard deviation.

^b^ CI: confidence interval.

^c^ All *P* values are adjusted for multiple comparisons.

^d^ ACV, LCV, and PCV were analyzed with the Wilcoxon signed-rank test, and the 95% CI could be calculated.

**Supplementary Material 6, Table**

**Table S2. Qualitative analyses of venous sinuses and venous plexuses.**

| **Venous plexus** | **Side** | **Posture** | **Venous blood ^a^** | | | **Intrarater reliability/Interrater reliability** |
| --- | --- | --- | --- | --- | --- | --- |
|  |  |  | **−** | **+** | **++** |  |
| Cavernous sinus |  | supine | 0 (0) | 20 (100) | 0 (0) | 1.00/1.00 |
|  |  | upright | 0 (0) | 20 (100) | 0 (0) | 1.00/1.00 |
| Vertebral plexus |  | supine | 0 (0) | 20 (100) | 0 (0) | 1.00/1.00 |
|  |  | upright | 0 (0) | 20 (100) | 0 (0) | 1.00/1.00 |
| Pterygoid plexus |  | supine | 3 (15.8) | 13 (68.4) | 3 (15.8) | 1.00/1.00 |
|  |  | upright | 4 (21.1) | 10 (52.6) | 5 (26.3) | 1.00/1.00 |
| Anterior condylar confluence | right | supine | 4 (20) | 10 (50) | 6 (30) | 1.00/1.00 |
|  |  | upright | 0 (0) | 5 (25) | 15 (75) | 1.00/0.727 |
|  | left | supine | 7 (36.8) | 6 (31.6) | 6 (31.6) | 0.839/1.00 |
|  |  | upright | 0 (0) | 9 (47.4) | 10 (52.6) | 0.783/1.00 |
| Vertebral artery venous plexus | right | supine | 4 (23.5) | 10 (58.8) | 3 (17.6) | 0.833/1.00 |
|  |  | upright | 0 (0) | 3 (17.6) | 14 (82.4) | 0.615/0.609 |
|  | left | supine | 6 (37.5) | 9 (56.3) | 1 (6.3) | 0.72/0.784 |
|  |  | upright | 0 (0) | 4 (25) | 12 (75) | 0.60/0.714 |
| Suboccipital cavernous sinus | right | supine | 4 (22.2) | 10 (55.6) | 4 (22.2) | 0.66/0.66 |
|  |  | upright | 0 (0) | 7 (38.9) | 11 (61.1) | 1.00/1.00 |
|  | left | supine | 6 (35.3) | 8 (47.1) | 3 (17.6) | 1.00/1.00 |
|  |  | upright | 1 (5.9) | 6 (35.3) | 10 (58.8) | 0.784/1.00 |
| Marginal sinus |  | supine | 8 (42.1) | 8 (42.1) | 3 (15.8) | 1.00/0.844 |
|  |  | upright | 2 (10.5) | 10 (52.6) | 7 (36.8) | 0.788/0.815 |
| Anterior internal vertebral venous plexus |  | supine | 9 (47.4) | 8 (42.1) | 2 (10.5) | 0.696/1.00 |
|  |  | upright | 3 (15.8) | 3 (15.8) | 13 (68.4) | 0.811/1.00 |

^a^ Numbers are presented as N (%). Venous plexuses and sinuses are classified as having poor (−), intermediate (+), or abundant (++) venous blood flow by visual reference.
